# Overactive EGF signaling promotes uv1 cell survival *via* increased phosphatidylcholine levels and suppression of SBP-1

**DOI:** 10.17912/micropub.biology.000266

**Published:** 2020-06-29

**Authors:** Matt Crook, Wendy Hanna-Rose

**Affiliations:** 1 Department of Science and Mathematics, Texas A&M University-San Antonio; 2 Department of Biochemistry and Molecular Biology, The Pennsylvania State University

**Figure 1:**
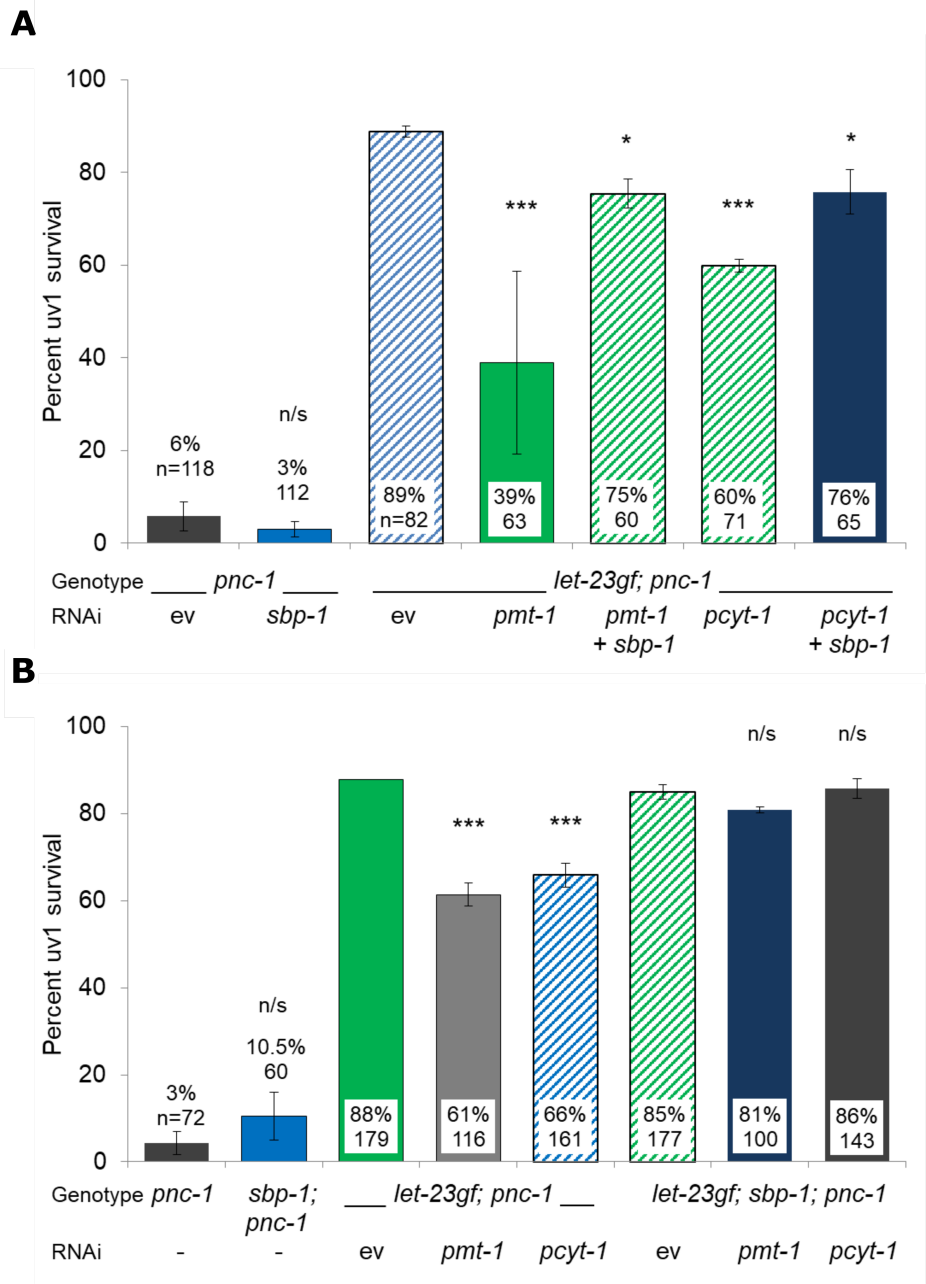
Overactive LET-23 signaling promotes uv1 survival *via* phosphatidylcholine synthesis and the suppression of SBP-1activity. A reduction in SBP-1 function by either a) RNAi or b) a *sbp-1(ep79)* hypomorph (Liang, *et al.*, 2010) reduces or eliminates the deleterious effect of reduced phosphatidylcholine synthesis from *pmt-1* or *pcyt-1* RNAi on uv1 cell survival. For a) uv1 cells were counted using an extrachromosomal *ida-1::gfp* marker, for b) an integrated *ida-1::gfp* marker was used. ev is RNAi empty vector L4440. Error bars are 1 S.D. with mean percentage uv1 survival, two repeats for a) and three repeats for b) and total number of animals scored. Mean uv1 survival was compared to that of control (ev or single mutant) animals by Fisher’s Exact Test; p < 0.05 = *, p < 0.001 = ***, p > 0.05 = n/s.

## Description

*Caenorhabditis elegans* uv1 cells undergo a dramatic cell death in a *pnc-1* NAD+ salvage deficient mutant (Huang and Hanna-Rose, 2006). uv1 cell death is a result of accumulation of the PNC-1 substrate nicotinamide (NAM), which overactivates the OCR-4 OSM-9 transient receptor potential cation V (TRPV) channel, causing excitotoxicity (Upadhyay, *et al.*, 2016, Vrablik, *et al.*, 2009). Reduced uv1 cell survival is almost completely restored in *pnc-1* mutant animals by sustained activation of the Epidermal Growth Factor (EGF) receptor LET-23, either by overexpression of the ligand LIN-3 or by a gain of function mutation in the receptor (Huang and Hanna-Rose, 2006). We presume that the constitutively activated receptor acts cell autonomously in the uv1 cells to promote survival, but have no direct evidence in support of that presumption, besides a prior report showing that a *let-23(sa62)* gain of function allele was able to induce uv1 specification in the absence of LIN-3 (Chang, *et al.*, 1999). To find genes required for uv1 survival in a *let-23(sa62gf);* *pnc-1(pk9605)* background, we performed an RNAi screen and found that phosphatidylcholine synthesis *via* PMT-1 or PCYT-1 is required for LET-23 to promote uv1 cell survival in *pnc-1* background (Crook, *et al.*, 2016). Moreover, treatment with exogenous phosphatidylcholine alone is partially sufficient to promote cell survival in the *pnc-1* mutant (Crook, *et al.*, 2016). The requirement of phosphatidylcholine synthesis indicates that membrane phospholipid composition, and by extension disruption of lipid homeostasis, may play a role in preventing TRPV-induced excitotoxic death.

Lipid synthesis homeostasis in general and phosphatidylcholine synthesis in particular are regulated by the *srebp-1* homolog *sbp-1* in *C. elegans* (Walker, *et al.*, 2011). SBP-1 is activated by low phosphatidylcholine levels resulting from *pmt-1* or *pcyt-1* RNAi (Walker, *et al.*, 2011). We hypothesized that uv1 survival in a *let-23gf:* *pnc-1* background decreases when SBP-1 is activated through reduced phosphatidylcholine synthesis *via* *pmt-1* or *pcyt-1* RNAi. We predicted that inactivation of SBP-1 when *pmt-1* or *pcyt-1* were knocked down by RNAi would restore uv1 survival to that seen in a *let-23gf:* *pnc-1* background.

We tested the role of SBP-1 by reducing its activity in a *let-23(sa62gf);* *pnc-1(pk9605)* background with reduced phosphatidylcholine synthesis. We took two complementary approaches. First we carried out double RNAi experiments (Ahringer, 2006) where both *sbp-1* and *pmt-1* or *pcyt-1* were knocked down in a *let-23(ga62gf);* *pnc-1(pk9605)* background using an extrachromosomal *ida-1p::gfp* marker. Each RNAi was carried out twice with an empty vector (L4440) as control. Double RNAi experiments were carried out by mixing equal volumes of overnight LB-Amp RNAi cultures before spotting NGM-Carb-Tet plates. We found that double RNAi of *sbp-1* with either *pmt-1* or *pcyt-1* resulted in a significant increase in uv1 survival compared with *pmt-1 or pcyt-1* RNAi alone (Fig. 1A). However, uv1 survival was not restored to the level seen in a *let-23(sa62gf);* *pnc-1(pk9605)* background, which may have been due to incomplete knockdown in the double RNAi experiments. To address this possibility, we created a *let-23(ga62gf)*; *sbp-1(ep79)*; *pnc-1(pk9605)* strain with an integrated *ida-1p::gfp* marker. We then knocked down *pmt-1* or *pcyt-1* in this background and compared its effect on uv1 survival to the same gene knockdowns in a *let-23(ga62gf)*; *pnc-1(pk9605)* strain, using an integrated *ida-1p::gfp* marker to count living uv1 cells. Each RNAi was carried out three times. We found that the *pmt-1* or *pcyt-1* knockdown induced reduction of uv1 survival in a *let-23(ga62gf)*; *pnc-1(pk9605)* background did not occur when these genes were knocked down in a *let-23(ga62gf)*; *sbp-1(ep79)*; *pnc-1(pk9605)* background (Fig. 1B). There was no effect of either *sbp-1* RNAi (Fig. 1A) or the *sbp-1(ep79)* mutant allele (Fig. 1B) alone on uv1 cell survival in a *pnc-1* mutant background. Thus, both of our approaches support the hypothesis that *sbp-1* is required for the reduction in uv1 survival seen when phosphatidylcholine synthesis is reduced in a *let-23(ga62gf)*; *pnc-1(pk9605)* background.

*let-23(sa62gf)* mediated uv1 survival requires phosphatidylcholine synthesis and reducing phosphatidylcholine synthesis reduces cell survival. Our work shows that the reduction in uv1 survival when *pcyt-1* or *pmt-1* are knocked down is dependent on SBP-1. We propose the following model based on these results and our previous work on TRPV-induced excitotoxic death (Upadhyay, *et al.*, 2016). An elevated level of NAM in a *pnc-1* mutant activates the OCR-4/OSM-9 TRPV channel and results in uv1 cell death. Constitutive activation of LET-23 promotes phosphatidylcholine synthesis *via* PMT-1 and PCYT-1. Elevated phosphatidylcholine levels result in a cell membrane in which the OCR-4/OSM-9 TRPV channel is nonfunctional, which in turn prevents its activation by elevated NAM levels and results in uv1 survival. Reduction of phosphatidylcholine levels *via pmt-1* or *pcyt-1* RNAi activates SBP-1, which restores lipid homeostasis and cell membrane phospholipid composition, resulting in a functional OCR-4/OSM-9 TRPV channel and cell death. Support for our model also comes from the observation that exogenous phosphatidylcholine restores OLQ survival in a *pnc-1* background, even though constitutive activation of LET-23 did not (Crook, *et al.*, 2016). The precise mechanism by which elevated phosphatidylcholine levels disrupt OCR-4/OSM-9 TRPV channel function is not yet known, but our work supports an interaction between phosphatidylcholine levels, ion channel function and SBP-1-mediated lipid homeostasis.

## Reagents

Strains:

HV560 *inIs179[ida-1p::gfp]* II; *pnc-1(pk9605)* IV

HV692 *let-23(sa62)gf* II; *pnc-1(pk9605)* IV; *Ex*[P*ida-1*p::*gfp 7.6*]

HV776 *let-23(sa62)gf inIs179[ida-1p::gfp]* II; *pnc-1(pk9605)* IV

HV790 *inIs179[ida-1p::gfp]* II; *sbp-1(ep79)* III; *pnc-1(pk9605)* IV

HV792 *let-23(sa62)gf inIs179[ida-1p::gfp]* II; *sbp-1(ep79)* III; *pnc-1(pk9605)* IV

*Ex*[P*ida-1*p::*gfp 7.6*] is from Zahn et al., 2001. None of these strains are or will be available at the CGC. The strains used in this study are available from the authors upon request.

We used the following clones from the Ahringer RNAi library: *pmt-1* *ZK622.3* II-4G04, *pcyt-1* *F08C6.2* X-3N20, and *sbp-1* *Y47D3B.7* III-6C01.
